# Two novel *VCP* missense variants identified in Japanese patients with multisystem proteinopathy

**DOI:** 10.1038/s41439-018-0009-7

**Published:** 2018-05-30

**Authors:** Michio Inoue, Aritoshi Iida, Shinichiro Hayashi, Madoka Mori-Yoshimura, Atsushi Nagaoka, Shunsuke Yoshimura, Hirokazu Shiraishi, Akira Tsujino, Yuji Takahashi, Ikuya Nonaka, Yukiko K. Hayashi, Satoru Noguchi, Ichizo Nishino

**Affiliations:** 1 Department of Neuromuscular Research, National Institute of Neuroscience, National Center of Neurology and Psychiatry, Tokyo, 187-8551 Japan; 2 Integrated Graduate School of Medicine, Engineering, and Agricultural Science, University of Yamanashi, Yamanashi, 409-3898 Japan; 3 Department of Clinical Genome Analysis, Medical Genome Center, National Center of Neurology and Psychiatry, Tokyo, 187-8551 Japan; 4 Department of Neurology, National Center Hospital, National Center of Neurology and Psychiatry, Tokyo, 187-8551 Japan; 5 Department of Neurology and Strokology, Nagasaki University Hospital, Nagasaki, 852-8501 Japan; 6 Department of Pathophysiology, Tokyo Medical University, Tokyo, 160-0023 Japan

## Abstract

*VCP* mutations were first associated with inclusion body myopathy with Paget’s disease of bone and frontotemporal dementia (IBMPFD) but was later associated with amyotrophic lateral sclerosis and Charcot–Marie–Tooth disease. Now, a new name, “multisystem proteinopathy (MSP)”, is proposed for this condition. *VCP* encodes valosin-containing protein, which is involved in protein degradation in the ubiquitin proteasome system. We report here two MSP patients with two novel heterozygous missense variants in *VCP*: c.259G>T (p.Val87Phe) and c.376A>G (p.Ile126Val).

## Data report

*VCP* mutations have been associated with (1) myopathy pathologically characterized by the presence of rimmed vacuoles, which is often called inclusion body myopathy (IBM), (2) Paget’s bone disease, or (3) frontotemporal dementia, or in some cases, all of these conditions. Therefore, the disease was collectively termed inclusion body myopathy with Paget disease of bone and frontotemporal dementia (IBMPFD)^1^. In the largest cohort studies of patients with VCP disease, myopathy was seen in 89% of patients, Paget’s disease of bone was seen in 43%, and dementia was seen in 30%^2^. The phenotype has now been expanded to amyotrophic lateral sclerosis (ALS) and Charcot-Marie–Tooth disease. *VCP* mutations account for 2% of familial ALS cases^3^. The prevalence of the disease is not known in Japan. However, as a referral center for muscle disease diagnosis, we have diagnosed 22 patients (20 families) with *VCP* mutations so far, including previous reports^4,5^. Due to the expanded phenotype, the term multisystem proteinopathy (MSP) is now proposed for a group of disorders that includes those associated with *VCP* mutations^6^. So far, 54 different mutations have been reported (Human Gene Mutation Database); however, a genotype–phenotype correlation has not been clearly established for most of these mutations, and the incidence, prevalence, penetrance, and history of VCP diseases have not been clarified. VCP is an essential AAA+ ATPase that is conserved in eukaryotes^7^. It is involved in major proteolytic pathways in cellular homeostasis, including membrane fusion, DNA damage repair, cell cycle and protein degradation. VCP forms a homo-hexamer with each monomer, comprising an N-terminal domain (NTD) and a pair of ATP domains, D1 and D2. A series of missense mutations, which mostly occur at the NTD-D1 interface, dominant-negatively cause specific malfunctions of protein homeostasis linked to degenerative disorders^8^. Thus, patient muscle pathology commonly shows cytoplasmic and nuclear protein accumulation in muscle fibers, together with rimmed vacuoles. Previously, we have reported seven Asian patients with VCP myopathy with/without bone and brain phenotypes^4^. The skeletal muscle pathologies indicated mixed neurogenic and myogenic changes, fibers with rimmed vacuoles, and the presence of cytoplasmic and nuclear inclusions. In this study, we report two novel heterozygous missense variants (c.259G>T (p.Val87Phe); c.376A>G (p.Ile126Val)) in *VCP* in two Japanese patients with MSP.

National Center of Neurology and Psychiatry (NCNP) is a referral center for neuromuscular diseases in Japan. Since 1978, we have performed pathological diagnoses on more than 18,000 muscle biopsies. For cases with undiagnosed hereditary muscle disease, we now perform mutation screening using an Ion PGM sequencer (Thermo Fisher Scientific, MA, USA) in combination with targeted gene panels that we recently developed to cover 187 known causative genes for hereditary muscle diseases in four panels: muscular dystrophy, congenital myopathy, metabolic myopathy, and myopathy with protein aggregations/rimmed vacuoles (myofibrillar myopathy [MFM] panel; Supplementary Table S1)^9^.

**Fig. 1:**
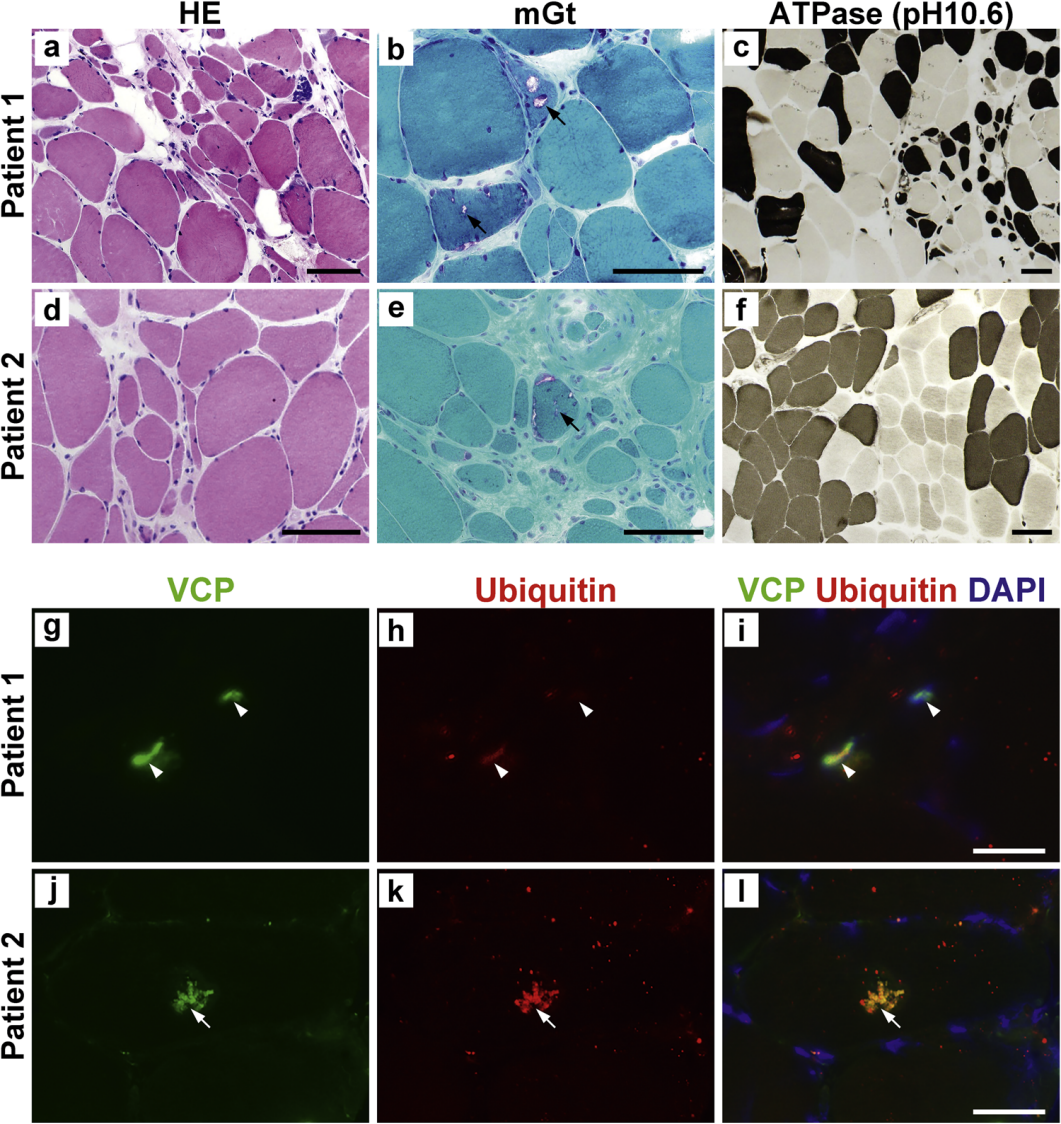
**a**–**f**: Histological analysis of the muscle biopsies from patients. **a**, **d**: Hematoxylin and eosin (HE), (**b**, **e**): modified Gomori trichrome (mGt), (**c**, **f**): ATPase (pH10.6). A group of atrophic fibers was observed in both patients (**a**, **c**, **d**, **f**). mGt staining revealed the presence of rimmed vacuoles (**b**, **e**, black arrows). Bar: 50 µm. **g**–**l**: Immunostaining for valosin-containing protein (VCP: **g**, **i**, **j**, **l**) and ubiquitin (**h**, **i**, **k**, **l**). VCP positive inclusions were co-stained with ubiquitin in both the nucleus (white arrowheads, DAPI positive) and the cytoplasm (white arrows, DAPI negative). Bar: 50 µm

Patient 1: The patient was a 73-year-old man with no family history of neuromuscular disease. He showed progressive muscle weakness and atrophy with involvement of the axial and proximal muscles. His symptoms started at the age of 71 as difficulty standing up. He also presented with psychological and cognitive impairment, including hallucinations and depression. His Mini-Mental State Examination and Frontal Assessment Battery scores were 26 and 11, respectively, suggesting mild cognitive dysfunction. He experienced difficulty in urination, suggesting autonomic dysfunction. He showed diminished deep tendon reflex, no pathologic reflex and no fasciculation. His serum CK levels were normal. There was no sign of bone involvement, e.g., elevated serum alkaline phosphatase (ALP) levels. A nerve conduction study showed reduced compound muscle action potential without evidence of slowed conduction velocities. Needle electromyography (EMG) showed decreased recruitment, suggesting neurogenic change. Brain single photon emission computed tomography showed diminished blood flow to the occipital lobe. MIBG myocardial scintigraphy revealed reduced uptake. Muscle biopsy showed mixed changes of myopathy and neuropathy (Fig. 1a–c). Scattered fibers with rimmed vacuoles and a few fibers with cytoplasmic bodies were seen (Fig. 1b,g–i). In addition, groups of small angular fibers and fiber type groupings were seen (Fig. 1a,c).

Patient 2: The patient was a 65-year-old man with no family history of　neuromuscular disease. In his late 40s, his initial symptom was difficulty raising his arms. Then, he developed progressive, mildly asymmetric upper limb-onset muscle weakness with facial involvement and scapular winging with sensory involvement. At 59 years old, he was unable to stand up without support. Over time, his respiratory function declined, and respiratory failure appeared at age 65 years, leading to death at age 66. His serum CK levels ranged from 246 to 669 U/L. No cognitive impairment was noted. He had no sign of bone involvement either in plain spinal X-ray or plain CT of the whole body. ALP testing data were not available. Needle EMG showed early recruitment, suggesting myogenic change. Muscle biopsy showed mixed changes, indicating myopathy and neuropathy (Fig. 1d–f) consisting of mild dystrophic change with scattered fibers with rimmed vacuoles (Fig. 1e,j–l), as well as group atrophy and fiber type grouping (Fig. 1d,f).

The clinical information and materials from patients were obtained for diagnostic purposes with written informed consent. All experiments in this study were approved by the Ethical Committee of NCNP. In both patients, a possibility of facioscapulohumeral muscular dystrophy 1 was excluded by long range PCR^10^.

**Fig. 2:**
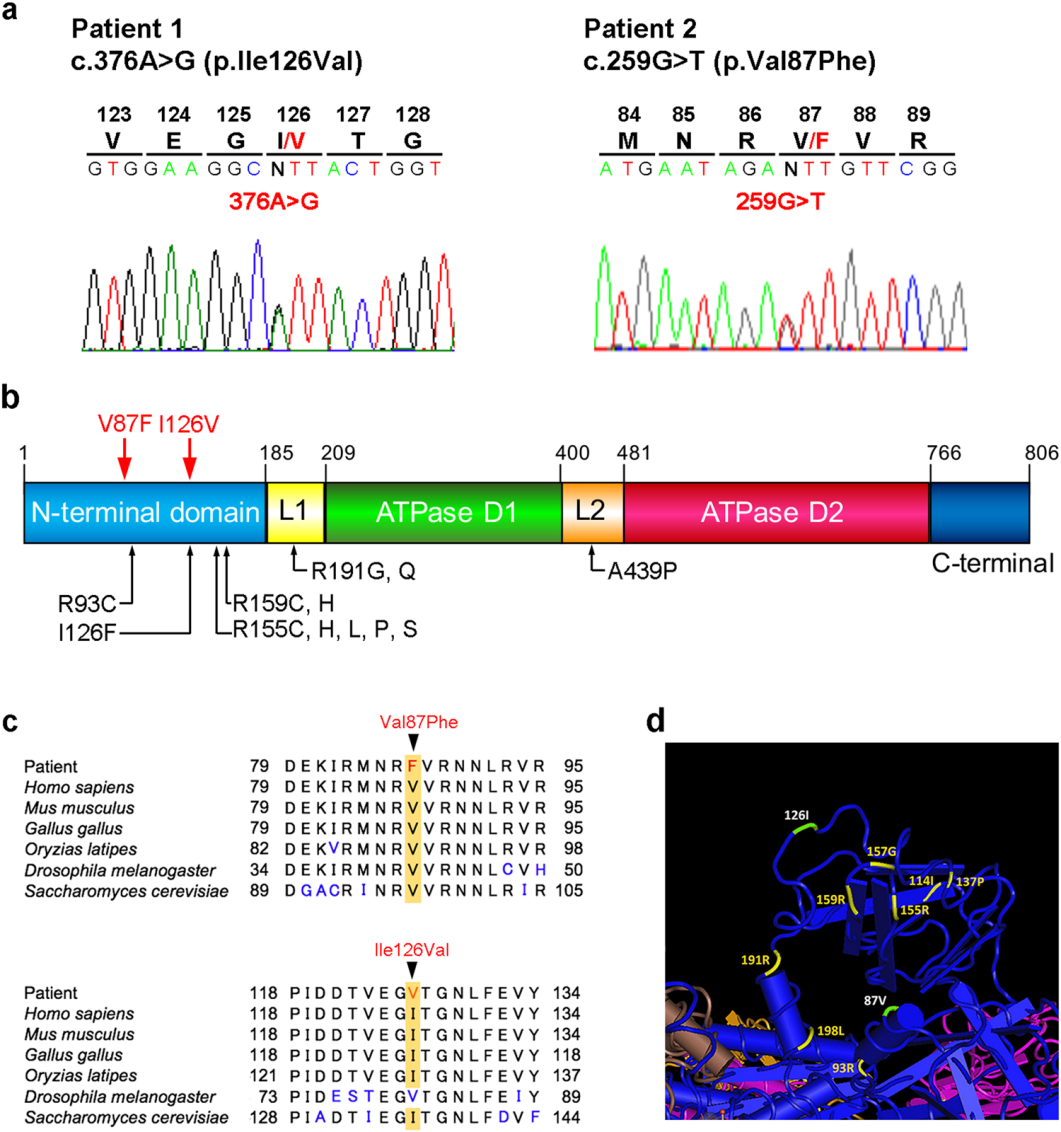
Results of valosin-containing protein (VCP) mutation screening. **a** Two novel heterozygous substitutions were identified (c.376A>G and c.259G>T). **b** Schematic functional domains and mutations of VCP. The locations of substitutions in this study are denoted by red arrows. Black arrows indicate the locations of previously identified mutations with inclusion body myopathy (IBM). **c** Multiple alignment of the VCP amino acid sequence in different species. The substitution sites in this study (Val87 and Ile126) are highly evolutionarily conserved. **d** The crystal structure of VCP protein showing the locations of the mutated residues detected in IBM. The crystal structure was obtained from the Molecular Modeling Database (MMDB ID: 82738). Our substitution sites (Val87 and Ile126) and those of previously reported mutations are indicated in white and yellow, respectively

Mutations in known causative genes for myopathy with protein aggregations/rimmed vacuoles were further screened in Patient 1 by an Ion PGM sequencer coupled with an MFM targeted gene panel. Coverage of the *VCP* locus was 100% at a depth of 20 reads. We identified a novel missense variant, c.376A>G (p.Ile126Val), within exon 4 in *VCP* (Fig. 2a). Follow-up sequencing was carried out and confirmed the variant. p.Ile126Val was located within the NTD, cofactor and ubiquitin binding function (Fig. 2b). The isoleucine residue was highly conserved from humans to yeast, except for in *Drosophila melanogaster* (Fig. 2c). Six of 10 mutation predictors showed the variant as “Deleterious” or “Disease causing” (Supplementary Table S2).

In Patient 2, the nucleotide sequences of the exons and exon/intron boundaries in *VCP* were further determined by the Sanger method based upon the clinical phenotype. A novel variant, c.259G>T (p.Val87Phe), in exon 3 was identified. The primer sequences for mutation screening were 5′-cagggctgctgcttactcc-3′ (forward primer) and 5′- ctgtaatgcaggctatctctgg-3′ (reverse). This mutated valine is also located within the NTD (Fig. 2b) and is evolutionally conserved (Fig. 2c). SIFT, PolyPhen-2 and Mutation Taster predicted the mutation to be “Tolerated” with a score of 0.14, “Possibly damaging” with a score 0.85 and “Disease causing” with a score of 0.99, respectively.

Finally, neither variant had been deposited in any databases, including dbSNP, 1000 Genomes, Exome Aggregation Consortium, Human Gene Mutation Database, Human Genetic Variation Database, Integrated Japanese Genome Variation Database or ClinVar (as the end of February 2018). Hence, the two variants were considered to be novel.

Cytoplasmic and nuclear inclusions and rimmed vacuoles, which are common pathological changes in VCP-related myopathy, were observed in muscles from our patients (Fig. 1g–l). These inclusions were positive for ubiquitin, TDP-43, SQSTM1 and VCP as previously reported^4^, suggesting again the impairment of ubiquitin-proteasome-degradation systems; consequently, autophagy induction is implicated in the pathomechanism based on the malfunction of mutated VCP in this disease.

In this study, we identified two novel variants, both of which cause amino acid substitutions. The variant in Patient 2, p.Val87Phe, was classified as “Possibly damaging” or “Disease-causing” in two of three predictions, and the residue is well conserved among species, suggesting that it is pathogenic mutation. In contrast, in the variant in Patient 1, the isoleucine of p.Ile126Val was replaced with valine in *Drosophila melanogaster*, suggesting that the mutation will be a relatively benign variant for pathogenesis. However, regarding variant p.Ile126Val, Patient 1 showed clinical symptoms, muscle pathology and protein aggregate patterns comparable to those in VCP disease. Six of 10 mutation predictors also showed the variant as “Deleterious” or “Disease causing” (Supplementary Table S2). Furthermore, it is to be noted that a different missense substitution at this amino acid residue (p.Ile126Phe) has been reported to be a pathogenic mutation^5^. Consequently, our clinical and *in silico* data suggest that p.Ile126Val is likely to be a milder mutation involved in the pathogenesis of VCP.

VCP is a homo-hexameric complex with each monomer comprising the NTD and a pair of ATPase domains (D1 and D2)^11^. As in almost all previous mutations, our substitution sites (Val87, Ile126) were also located in NTD and were conformationally aligned nearby (Fig. 2b,d). Fine structural analyses of full-length VCP or nucleotides in the NTD-D1 domain by crystalography^12^ and NMR spectroscopy^13^ unraveled that the reported missense mutations in NTD had roles in the malfunction of VCP molecules. Based on these analyses, the importance of dynamic structural changes around the Val87 site for disease pathogenesis has been discussed. Further analyses of genetic variations, structural changes and the ATPase activity of mutated VCP and the resulting cellular and muscle phenotypes will improve our understanding of the pathomechanism of VCP diseases and provide insights into the development of therapies.

## Electronic supplementary material

Supplementary Table S1

Supplementary Table S2

## Data Availability

The relevant data from this Data Report are hosted at the Human Genome Variation Database at 10.6084/m9.figshare.hgv.2312, 10.6084/m9.figshare.hgv.2315.
